# Statistics of Cancer in the Breast

**Published:** 1880-07

**Authors:** 


### STATISTICS OF CANCER IN THE BREAST.

DR. J. OLDEKOPP has published in the twenty-fourth volume of the *Archiv fuer Klinische Chirurgie*, a statistical summary of all cases of mammary cancer occurring in Professor Esmarch's hospital and private practice from 1850 to 1878. With regard to age, most of the cases occurred between the forty-eighth and fiftieth years; in 123 patients, the age did not exceed 48; in 71, it was between 48 and 58; and, in 35, the age was 59 and upwards. In 21 cases there are no particulars as regards age. Women who had borne more than six children furnished the greatest contingent, and next came those who had no children. There were 9 in this category, against 103 who had given birth to children. In 61 cases in which the information could be obtained, 15 had not, and 46 had, suckled their children. In 36 cases mastitis had preceded; but in only 9 was it ascertained with certainty that the cancer had its starting point in an induration or cicatrix remaining after the mastitis. In 3 cases there had been contusion with extravasation; the extravasation after some years, forming the centre of the new growth. In two cases the seat of the primary nodule was a part of the breast which had for some years been pressed on by the string of a corset; in a third, it was a part that was often pressed on by a yoke. In 126 cases the right breast was diseased, in 102 the left. The outer and upper part of the mamma was most frequently first affected; and this is ascribed by Dr. Oldekopp to the greater liability of this part to injury. In three cases the cancer was preceded by chronic eczema of the breast. Circumstances indicating the influence of hereditary tendency were noticed in eleven cases. The average duration of life from the commencement of the disease was, in the cases not operated on, 22.6 months; in those operated on 38.1 months. On 225 patients 287 operations were performed. Of these 225, there died in the hospital 28; viz., 5 from return of the cancer, and 23 from the operation; among these were 14 cases of total extirpation of the mamma with removal of the axillary glands. With regard to the influence of treatment on the mortality and on the time required for healing. Dr. Oldekopp's statistics show no marked difference between the antiseptic and the non-antiseptic methods; he remarks, however, that erysipelas has been less frequent in Dr. Esmarch's practice since the introduction of the antiseptic method. The time after the operation at which the disease returned is noted in 112 cases. In 14 cases it immediately followed the operation; in 15 it took place within the first month; in 23 within three months; in 15 within more than three and less than six months; in 13 from the seventh to the ninth month; in 14 from the tenth to the twelfth month; in 9 from the thirteeth to the eighteenth month; and in 8 within three years. In one doubtful case the interval is said to have exceeded three years. At the time of the report 44 of the women had remained free from a return of the disease; of these six had died of intercurrent diseases; three within three years since the operation, and three after three years. In 15 the time during which they had remained free from relapse was under three years; and, assuming three years as the extreme time for a return of the disease, 26 could be regarded as definitely cured; in 10 of these the infiltrated axillary glands had been removed with the mammary cancer. In some cases a second operation was necessary. Although the number of cases in which a complete cure followed the operation is not so large, Dr. Oldekopp regards it a sufficiently encouraging to induce surgeons to operate early, and thus increase the chance of good result.—*British Medical Journal.*

